# Germ cell specification and ovary structure in the rotifer *Brachionus plicatilis*

**DOI:** 10.1186/2041-9139-1-5

**Published:** 2010-08-02

**Authors:** James M Smith, Andrew G Cridge, Peter K Dearden

**Affiliations:** 1 Laboratory for Evolution and Development, Genetics Otago and the National Research Centre for Growth and Development, Biochemistry Department, University of Otago, PO Box 56, Dunedin, Aotearoa-New Zealand; 2 Institute of Natural Sciences, Massey University Albany Campus, Private Bag 102904, North Shore MSC, Auckland, Aotearoa-New Zealand

## Abstract

**Background:**

The segregation of the germline from somatic tissues is an essential process in the development of all animals. Specification of the primordial germ cells (PGCs) takes place via different strategies across animal phyla; either specified early in embryogenesis by the inheritance of maternal determinants in the cytoplasm of the oocyte ('preformation') or selected later in embryonic development from undifferentiated precursors by a localized inductive signal ('epigenesis'). Here we investigate the specification and development of the germ cells in the rotifer *Brachionus plicatilis*, a member of the poorly-characterized superphyla Lophotrochozoa, by isolating the *Brachionus *homologues of the conserved germ cell markers *vasa *and *nanos*, and examining their expression using *in situ *hybridization.

**Results:**

*Bpvasa *and *Bpnos *RNA expression have very similar distributions in the *Brachionus *ovary, showing ubiquitous expression in the vitellarium, with higher levels in the putative germ cell cluster. *Bpvas *RNA expression is present in freshly laid eggs, remaining ubiquitous in embryos until at least the 96 cell stage after which expression narrows to a small cluster of cells at the putative posterior of the embryo, consistent with the developing ovary. *Bpnos *RNA expression is also present in just-laid eggs but expression is much reduced by the four-cell stage and absent by the 16-cell stage. Shortly before hatching of the juvenile rotifer from the egg, *Bpnos *RNA expression is re-activated, located in a subset of posterior cells similar to those expressing *Bpvas *at the same stage.

**Conclusions:**

The observed expression of *vasa *and *nanos *in the developing *B. plicatilis *embryo implies an epigenetic origin of primordial germ cells in Rotifer.

## Background

The segregation of the germline from somatic tissues is an essential process in the development of all animals. Despite this, specification of the progenitors of the germline, the primordial germ cells (PGCs), takes place via two broadly different strategies across animal phyla (reviewed in [1]). Germ cells can be specified early in embryogenesis by the inheritance of maternal determinants inherited in the cytoplasm of the oocyte ('preformation'), as in *Drosophila*, *Danio rerio*, *Xenopus laevis *and *Caenorhabditis elegans*. Alternately, as in the mouse, germ cells can be selected later in the embryonic development from undifferentiated precursors by a localized inductive signal ('epigenesis'). While the majority of genetic model organisms specify germ cells by preformation, epigenesis is the more prevalent mechanism for PGC specification across animal phyla. This, along with the prevalence of epigenesis for germline specification in basal metazoans, implicates epigenesis as the ancestral mechanism of germ line specification in animals [2]. It should be noted, however, that the majority of studies of PGC specification are from two of the three animal super-phyla as determined by modern phylogenetics [3], namely the Ecdysozoa and the Deuterostoma. Relatively little is known about how the germline is specified in the Lophotrochozoa, which is the largest (containing more than half of all animal phyla [4]) and exhibits the greatest diversity in body plans of the three superphyletic groups of animals.

Despite these broadly different mechanisms for the specification of the germline, some of the proteins involved in PGC specification are highly conserved and expressed in germ cells whether they form by epigenesis or preformation. PGC specification can thus be reliably tracked by the expression of germline markers such as the products of the *vasa *and *nanos *genes (reviewed in [1]).

*vasa *encodes an adenosine triphosphate (ATP)-dependent RNA helicase that is a member of the DEAD box protein family [5,6]. In *Drosophila*, in which *vasa *expression and function has been best characterized, Vasa protein is associated with polar granules [7], electron-dense structures within the oocyte pole plasm which give rise to the germline, and is essential for formation of the pole plasm and progession of oogenesis. On a molecular level, Vasa acts as a translational regulator of the oocyte-specific maternal mRNAs, such as *gurken*, through binding of the translation factor eIF5B [8] and is also required for the localization of *nanos *RNA [9]. Throughout animals *vasa *is expressed in germ cell progenitors and other stem cell types [1], regardless of the mechanism of germline specification. In mice, the best characterized species that undergoes epigenesis, *vasa *homologues are expressed in PGCs and are required for development of the male germline [10]. *Vasa *RNA and protein have been found associated with PGC specification and development in many species across the metazoa [11-20]. While the precise molecular function of *vasa *across evolution remains elusive, the broad conservation of germline components with which *vasa *interacts in *Drosophila *suggests that the mode of action of *vasa *is conserved across germline development in animals.

*nanos *genes have been implicated in specification of both germline and somatic cell fate, although their roles in the formation and/or maintenance of PGCs are more broadly conserved [21] and are considered to be their ancestral function. Nanos proteins contain two highly conserved C-terminal CCHC zinc finger domains and act as translational inhibitors [22], repressing somatic cell fate in the developing germline in *Drosophila*. Roles for Nanos in the development of somatic tissues have been identified in insects, most notably in establishing embryonic polarity [22], and, more recently, in a small number of Lophotrochozoan phyla [18,23-25] where they have been implicated in specification of somatic cell lineages such as mesoderm.

In this study we examine the expression of *vasa *and *nanos *in germline development of the monogonont rotifer *Brachionus plicatilis*. The rotifera are a diverse non-segmented aquatic lophotrochozoan phyla containing two major branches, the bdelloids and the monogononts. Bdelloids are obligately asexual while monogononts are facultatively sexual, producing either amictic (diploid) or mictic (haploid) offspring. The ovary of the monogonont rotifer consists of a syncytial vitellarium connected via an oviduct to a germarium containing primordial germ cells. Cytoplasmic bridges connect developing oocytes to the vitellarium, through which maternal factors synthesized in the vitellarium are transported [26]. Morphological investigations have suggested rotifer PGCs are produced by a preformation mechanism ([27]; referenced in [1]), though this has yet to be confirmed by molecular analyses.

## Results

### *Brachionus *ovary anatomy and the origin of oocytes

**Figure 1:**
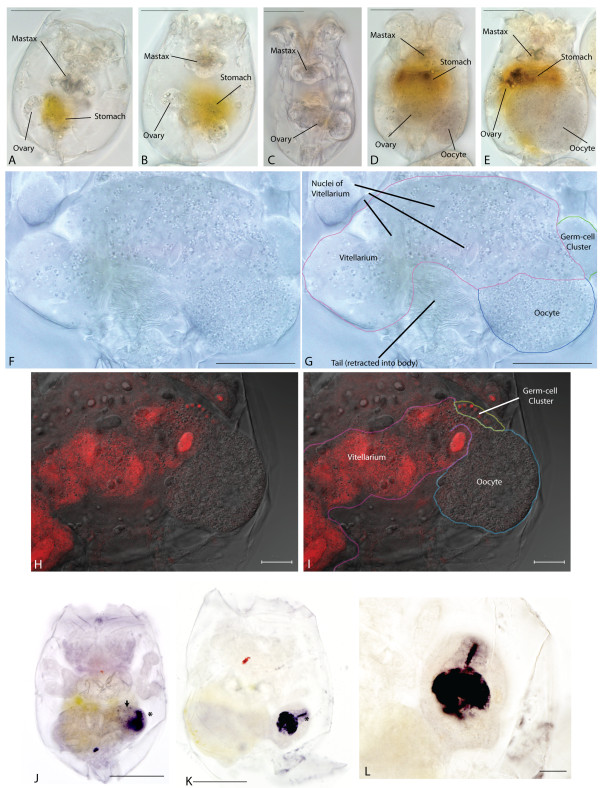
**Structure of the *Brachionus plicatilis *ovary**. Light and fluorescent images of the *Brachionus *ovary. (A-E) Differential interference contrast (DIC) images of whole *Brachionus *indicating the ovary and other morphological structures. Scale bars indicate 100 μm. Rotifer range from starved adult individuals with greatly reduced ovaries (A) to normal individuals (C), to those with a developing oocyte (D) to those with a large oocyte deforming other structures (E). (F and G) Higher magnification image of a *Brachionus *ovary (annotated in G) under DIC optics showing the structures of the vitellarium, germ cell cluster and oocyte. Scale bars represent 50 μm. Note: the granules/lipid droplets in the vitellarium and their concentration in the oocyte. (H and I) High magnification mixed confocal/phase contrast image of a *Brachionus *ovary stained with propidium iodide (red) for nuclei (annotated in I). All of the cell types of the ovary are visible. Scale bars represent 50 μm (J-L) Expression of compounds reactive to DAB and H_2_O_2, _producing intense black staining. Staining is in the oocyte and in a tube (asterisks in J and K), passing near the germ-cells, that appears to connect the vitellarium with the developing oocyte. Granular staining is also seen in the vitellarium (arrowhead in J) Scale bars in J and K are 100 μm, 50 μm in L.

The ovary of *Brachionus *females lies in the posterior half of the animal and is regulated by nutritional status. Starved rotifers have reduced ovaries that condense towards the midline of the animal (Figure 1A and 1B). Fully fed, reproductively active rotifers have large ovaries (Figure 1C-E) with clearly visible oocytes. The *Brachionus *ovary is made up of three major populations of cells (Figure 1F-I). The vitellarium, a large, nutritionally-sensitive structure, is thought to be syncytial [26]. The vitellarium is distinguished, in well-fed animals, by large elongate nuclei. Attached to the side of the vitellarium is typically the developing oocyte, a large cell with a small rounded nucleus. This cell, the oocyte, expands in volume over time, often growing to such an extent before laying that it deforms the structure of the ovary, displacing the nuclei of the vitellarium, as well as pushing other internal organs of the rotifer towards the head. The developing oocyte always forms on one side of the ovary, anterior and to the left when the rotifer is viewed with ovary uppermost (that is, lying over the gut). The adjoining region of the vitellarium also harbours a population of small cells (usually 20-22 in newly hatched adult rotifers), arranged in rows, with small rounded nuclei, that we, and others [26,28], take to be the precursors of the oocyte; the PGCs.

The cytoplasm of the developing oocyte and that of the vitellarium is granulose, probably due to lipid droplets, when viewed under differential interference contrast (DIC) or phase contrast optics. These droplets are concentrated in the oocyte (Figure 1F-I). We interpret this to mean that these droplets are synthesized in the vitellarium and are then transported into the developing oocyte via cytoplasmic bridges.

*Brachionus *oocytes express a factor that reacts strongly with DAB and hydrogen peroxide, staining darkly (Figure 1J-L). It is not clear what this factor is, other than it has putative peroxidase activity, but it is distributed in an interesting pattern suggesting that it may too be transported into the oocyte. Peroxidase-substrate-positive granules are distributed sparsely throughout the cytoplasm of the vitellarium (arrow in Figure 1J), increasing in density nearest the developing oocyte, and staining is particularly intense in a short tube or column leading to the developing oocyte from the vitellarium, flanked by the PGCs (asterisks in Figures 1J and 1K). The cytoplasm of the oocyte itself also stains intensely for this factor. This evidence strongly implies that the vitellarium, with its large active nuclei, makes a number of factors - both RNA, protein and lipid droplets - that are transported into the cytoplasm of the maturing oocyte and it may be this accumulation of maternal products transported from the vitellarium that swells the egg, eventually causing deformation of the vitellarium, before the egg is finally released.

### Identification of Rotifer *vasa *and *nanos*

In order to better describe the rotifer ovary and the origins of germ cells during development we isolated orthologues of *vasa *and *nanos*, both conserved germ cell markers.

**Figure 2:**
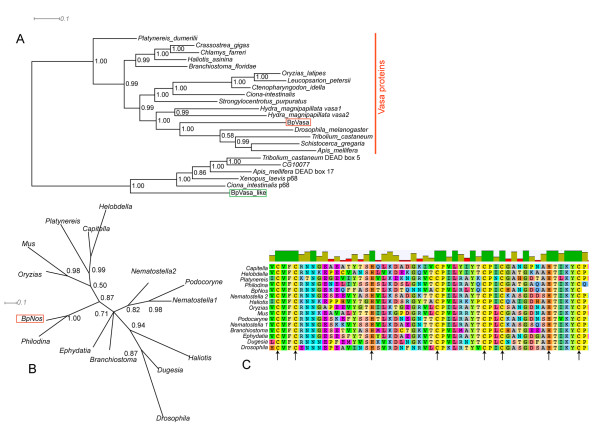
**Phylogenetic analyses of *Brachionus *Vasa and Nanos related predicted protein sequences**. (A) Rooted phylogenetic tree, produced using Bayesian approaches, of Vasa and related proteins. Two Vasa-like DEAD box RNA helicases from *Brachionus *are analysed with Vasa and p68-like sequences from animals. BpVasa (red box) groups with Vasa sequences from other animals, against p68-like sequences (with which the tree is rooted); BpVasa-like (green box) clusters with p68-like sequences. (B) Unrooted phylogenetic analysis of Nanos-like protein sequences including BpNos. BpNos clusters closely with Nos from *Philodina*, a bdelloid rotifer with a posterior probability of 1. As there are no closely related sequences to Nos in animal genomes with which to root this tree we also examined the protein alignment (C) for the highly conserved residues of the Nos zinc-finger domains (arrows) all of which are present.

*Brachionus *sequences similar to *vasa *were identified and assembled from sequencing of degenerate PCR fragments, published EST data and a pyrosequencing transcriptome sequencing project carried out using 454 technology. Sequences similar to *vasa *were assembled using CAP3 and predicted protein coding sequences obtained. By Blast analysis, these identified sequences showed similarities to Vasa proteins from other lophotrochozoan species. Bayesian phylogenetic analysis using Vasa protein sequences as well as protein sequences similar to the closest DEAD box RNA helicase genes identified in our 454 transcriptome data (Figure 2A) indicates that this *Brachionus vasa*-like sequence clusters with high posterior probability with other metazoan Vasa proteins, to the exclusion of closely related DEAD box sequences. This implies that the sequence we have identified is a *Brachionus *orthologue of Vasa. We designate the gene encoding this protein *Bpvasa (Bpvas)*.

*nanos *genes encode zinc finger transcription factors with a highly conserved DNA binding domain. Blast searches of available *Brachionus *sequence data, as well as a fragment of sequence obtained via degenerate PCR, identified a small number of sequences with similarity to *nanos*. These sequences were assembled using CAP3 and a predicted protein coding sequence obtained. Alignment of this protein sequence with those of other metazoan Nanos proteins (Figure 2C) showed our putative *nanos *homologue encoded a strongly conserved zinc finger binding domain with two characteristic CCHC motifs (arrowed). Similarity outside of this region was limited (data not shown) and so Bayesian phylogenetic analysis was performed on an alignment of just the zinc finger domain. The resulting consensus tree (Figure 2B) shows that this *Brachionus *sequence is very similar to other Nanos proteins and clusters most closely with Nanos protein from the Bdelloid rotifer *Philodina*. This analysis, and the conserved residues, strongly indicates that we have identified a *Brachionus *homologue of *nanos*. We designate the gene encoding this protein *Bpnanos (Bpnos)*.

### Gene expression in the Rotifer ovary

As *vasa *and *nos *are conserved germ cell markers we examined the distribution of RNA expression of the *Brachionus *orthologues of these genes in the adult ovary.

**Figure 3:**
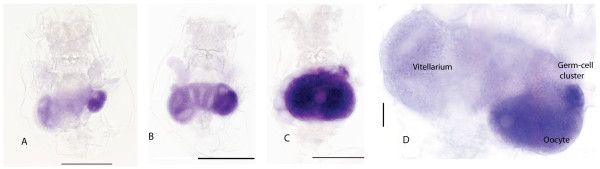
**Expression of *Bpvas *RNA in the *Brachionus *ovary**. (A-C) Expression of *Bpvas *RNA at three stages of oocyte provisioning. *Bpvas *RNA (blue) is present at low levels in the vitellarium and at higher levels in the germ-cell cluster and higher still in the oocyte. Scale bars indicate 100 μm, anterior to the top. (D) Magnification of an ovary showing *Bpvas *expression. Scale bar is 50 μm.

*Bpvas *RNA is expressed in all the cell types of the ovary, but at different levels (Figure 3). Expression is weak in the vitellarium but ubiquitously distributed. Expression is higher in the putative germ-cell cluster. As the oocyte begins to develop (Figure 3A-C, close-up in 3D) *Bpvas *RNA comes to be most highly expressed in the oocyte with very high levels present in oocytes that are about to be released. No *Bpvas *expression is seen in any other tissue of adult rotifer.

**Figure 4:**
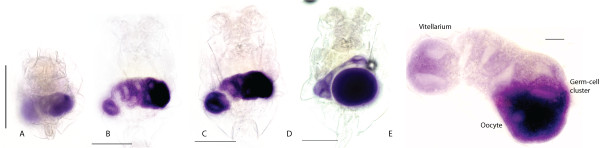
**Expression of *Bpnos *in the *Brachionus *ovary**. (A-D) *Bpnos *RNA expression at four stages of oocyte provisioning. *Bpnos *RNA expression is present at slightly lower levels in vitellarium than in the germ cells and oocytes. No expression can be seen outside of the ovary. Scale bars indicate 100 μm, anterior to the top. (E) Magnification of an ovary showing *Bpnos *expression in the germ cell cluster and oocyte with lower levels in the vitellarium. Scale bar indicates 50 μm, anterior to the top.

*Bpnos *RNA expression has a very similar distribution in the ovary to that of *Bpvas *(Figure 4). *Bpnos *RNA is present ubiquitously in the vitellarium with higher levels in the putative germ-cell cluster. As the oocyte develops and matures *Bpnos *RNA expression becomes stronger in the oocyte and germ cell cluster (Figure 4B-D). No *Bpnos *RNA expression is seen in any other adult rotifer tissue. Control staining with sense RNA probes from *Bpnos *or *Bpvas *shows no staining in adult or embryonic tissues (data not shown). *In-situ *hybridization using the method described here using probes for *Brachionus pax6 *RNA gives the same expression pattern as that reported in [29] (data not shown).

### *vasa *and *nanos *expression in the developing Rotifer embryo

**Figure 5:**
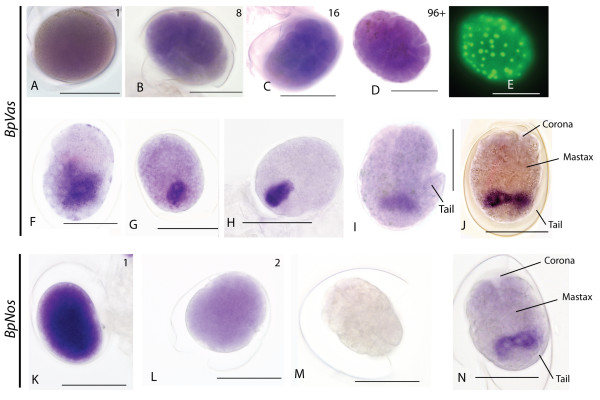
***Bpvas *and *Bpnos *expression in *Brachionus *embryos**. (A-J) Expression of *Bpvas *RNA in *Brachionus *embryos. Scale bars are 100 μm. Where known, anterior is at the top. (A) *Bpvas *RNA is first seen ubiquitously in the just-laid egg and continues to be expressed in all cells through eight (B) and 16 (C) cell stages up to stages where we can count at least 96 cells (D and E). At stages later than that, *Bpvas *expression is lost from most cells (F) and focuses to a small number of cells (G and H), which become partitioned into the posterior of the embryo into a dumbbell-shaped domain just anterior to the tail, which we interpret as the developing ovary. (K-N) Expression of *Bpnos *RNA. All scale bars are 100 μm, where known anterior is to the top. (K) Expression of *Bpnos *RNA in a one-cell embryo; RNA is ubiquitous throughout the embryo. This RNA is substantially reduced in two-cell embryos (L) and is absent by the 16-cell stage (M) throughout the rest of development until embryos are just about to hatch (N) when expression appears in a similar dumbbell-shaped domain as emerges for *Bpvas *expression.

In order to understand the origins of germ cells in *Brachionus *we also examined the distribution of *Bpnos *and *Bpvas *RNA in developing embryos. *Bpvas *RNA expression remains present in just-laid, 1 cell embryos; probably persisting maternal RNA (Figure 5A). Expression remains ubiquitous in embryos (Figure 5A-E) until at least 96 cells (as judged by nuclear staining; Figure 5E) are present. After this stage, expression narrows to a small cluster of two to six cells at one end of the embryo (Figure 5F-H). This narrowing appears to be due to loss of RNA expression from the majority of cells (Figure 5F). As development continues this cluster of *Bpvas *positive cells remains located towards one end of the embryo and, as morphology becomes apparent, it is clear that this is the posterior end (Figure 5I). As the corona and mastax become visible just before hatching, *Bpvas *expression is present in a dumb-bell shaped domain in the posterior in a region consistent with the developing ovary (Figure 5J).

*Bpnos *RNA expression is also present in just-laid eggs (Figure 5K) but expression is significantly reduced by the two-cell stage (Figure 5L) and absent by the 16-cell stage. *Bpnos *RNA expression is not present in the animal until just before hatching; the RNA is located in a dumb-bell shaped patch of cells, similar to that seen for *Bpvas *staining, in the posterior regions of the embryo, probably representng the developing ovary (Figure 5N).

## Discussion

### Germ cell development in *Brachionus plicatilis*

The expression of *vasa *and *nos *in the developing *B. plicatilis *embryo appears consistent with an origin of primordial germ cells via epigenesis in rotifer. There exists no specific subpopulation of embryonic cells showing the expression of both factors from early in development, nor is there any evidence for the germline-specific localization of *Bpvas *or *Bpnos *RNA in the egg or embryo until later stages. Both of these findings indicate that preformation of PGCs is unlikely in *Brachionus*; *Bpvas *expression is ubiquitous throughout the early embryo, while *Bpnos *expression disappears from the embryo beyond the four-cell stage and only reappears beyond the 96-cell stage in a posterior cluster of cells which we take to be the same subset of cells marked by *Bpvas *expression. The observed expression pattern of *Bpvasa *RNA in *B. plicatilis*, whereby the broad maternal distribution of expression throughout the early embryo becomes restricted to putative germline precursors as development progresses, is consistent with that in annelid Lophotrochozoans such as *Platynereis *[18], *Capitella *[23] and *Tubifex *[30].

It would seem probable that some form of inductive event or process maintains *Bpvas *expression in these cells as it is lost from the rest of the embryo and/or induces the expression of *Bpnos *in the same cells (it is not possible at present to dissect which, if either, of these events precedes the other). Based on the maintenance of *Bpvas *expression throughout the embryo up to this point, it would seem that all cells retain the ability to become PGCs, though only a subset do, presumably under the influence of some form of positionally specified epigenetic signal.

Our observation of an origin for PGCs via epigenesis in rotifers contrasts with the historical literature, which proposes PGCs are preformed prior to gastrulation ([27]; referenced in [1]). It should be noted, however, that these studies were based on morphological characterization of cell types by light microscopy. This is the first experimental investigation of rotifer PGC formation by way of molecular evidence, in particular expression profiling of conserved germ cell markers during embryogenesis. At this stage, we cannot entirely rule out preformation as a mechanism for forming PGCs in rotifer, due to the possibility that post-translational regulation could provide a means by which Vasa and/or Nos proteins could be localized to specific cell types within the oocyte throughout embryonic development - despite the the ubiquitous mRNA distribution of *vasa *and the absence of *nos *transcripts at these stages. Post-translational regulation of proteins involved in oogenesis is a recurring theme in *Drosophila *(reviewed in [9]). Despite this we see no direct evidence to support preformation of PGCs in this rotifer. Our findings are consistent with the observation that epigenesis is the most common and likely to be the ancestral means of generating germ cells amongst the Lophotrochozoa [1], with only a subset of annelid, mollusc and platyhelminth species reported to produce PGCs via preformation.

### Absence of somatic cell expression of *vasa *and *nos*

Expression of both *Bpvas *and *Bpnos *appears largely absent from somatic cell lineages in late embryonic and adult rotifer, save for expression in the vitellarium of the ovary associated with 'loading' of the developing oocyte with maternal transcripts. Indeed, *Bpnos *transcripts are absent throughout the development of the rotifer, other than very early in embryonic development, (presumably maternally derived) and in developing PGCs of the ovary. This is in contrast to several Lophotrochozoan species in which *nos *is expressed in somatic cell lineages and has roles in early embryonic patterning, predominantly of mesoderm [18,23-25]. Rotifer are known to differ from other Lophotrochozoan phyla in key elements of early embryogenesis; while rotifer are said to exhibit 'modified' spiral cleavage [31] they lack the heavily stereotyped early cell division pattern of canonical spiral cleavage (Spiralia), such that characteristic cells of spiral cleavage are not evident [29]. For instance, immunohistochemistry for activated MAPK (mitogen-activated protein kinase) which marks the 4d micromere, the mesodermal organizer in spiralian lophotrochozoans, [32] fails to identify an equivalent structure in rotifer (data not shown), which suggests that mesodermal specification may take place via alternative mechanisms than those identified in spiralian phyla.

The expression patterns of *Bpvas *and *Bpnos*, as well as the localization of lipid droplets/vesicles and reactivity to DAB, suggests that the vitellarium acts to nutritionally provision the oocyte. The observation of *Brachionus *oocytes expressing a factor that reacts strongly to peroxidase substrates is interesting in light of the report of expression of a plant-like peroxidase gene in *Hydra *oogenesis [33], although no homologue to this Hydra gene (*HvAPX1*) was detected in our *Brachionus *transcriptome sequence (data not shown). Our observation that the size of the rotifer ovary is responsive to nutrition suggests that it is a highly metabolically active tissue that, in adverse conditions, is too expensive to maintain. It is possible, however, that is responding to more subtle changes in diet, such as macronutrient status [34] or amino-acid balance [35]. The vitellarium transports both RNA and protein in the oocyte (as well as the lipid vesicles) and appears to maintain a cytoplasmic bridge to the oocyte until the egg is ejected. None of the expression patterns of RNA we have examined suggests that the vitellarium acts to pattern the early egg, although the asymmetry in the localization of the egg, with respect to the vitellarium and ovary in the adult, implies that the provision of patterning data is possible.

We have described, for the first time in molecular detail, the activity of the *Brachionus *ovary and the localization and formation of germ cells by epigenesis in the developing *Brachionus *embryo. How the expression of *Bpvas *RNA is constrained to a few cells at the putative point of germ cell specification, and how these cells then come to be recruited to the ovary remains to be discovered. Indeed little is known about the molecular, or even morphological, aspects of development in Rotifer but *Brachionus*, with its fast generation time and accessible embryos, is likely to be an excellent model system for studying development in this phylum.

## Methods

### Rotifer culture

*B. plicatilis *Nevada (Additional File 1) rotifers were cultured at 25°C in conical flasks in F2 media [36,37] made with Instant Ocean artificial seawater (Aquarium Systems, OH, USA) and fed either *Duniella *sp. microalgae or Culture Selco High Density commercial rotifer feedstock (2g/L; INVE Aquaculture, UT, USA).

### Identification of *B. plicatilis vasa *and *nanos *sequences

Fragments of *B. plicatilis vasa *and *nos *genes were initially amplified from cDNA by degenerate polymerase chain reaction (PCR). Rotifers were concentrated by centrifugation, total RNA was extracted using Trizol reagent (Invitrogen) and first-strand cDNA synthesis carried out with Superscript III (Invitrogen, CA, USA) using an oligo-dT primer. Degenerate PCR primers were used to amplify the conserved domains of the *vasa *and *nanos *genes from *B. plicatilis *cDNA. The sequences of primers used were: vasa-F2 5'-GA(A/G)AAICCCAT(A/G)TCIA(A/G)CAT-3' and vasa-F2 5'-CAGACGGGITCIGGIAA(A/G)AC-3' [38]; nanos1 5'-CGGAATTCCGTG(C/T)GTITT(C/T)TG(T/C)(G/A/C)AGIAA(C/T)AA-3' and nanos2 CGGGATCCCGGG(G/A)CA(G/A)TA(T/C)TTIA(T/C)IGT(GA)TG-3' [39]. PCR products were cloned into pCRII-TOPO (Invitrogen) for transformation into *E. coli*, sequencing and the maintenance of clones.

A modified form of 3' RACE was used to amplify an additional 3' sequence of *B. plicatilis nanos *sufficient for it to be used as a template for *in situ *hybridization probe synthesis. Rotifer cDNAs were directionally cloned into the Invitrogen CloneMiner cDNA Library Construction system, pooled library plasmid DNA from which was used as a template for PCR amplification using the nanos 5' primer and a vector reverse primer. This PCR product was cloned and sequenced. A new 3' primer was designed in order to complement the very 3' end of the *Bpnos *transcript, which was used in conjunction with the 5' primer in order to amplify, clone and sequence this fragment.

Sequences obtained were subsequently compared with ESTs made available in the National Center for Biotechnology Information databases from *B. plicatilis *EST sequencing projects [40,41] to confirm the integrity and continuity of sequences obtained by degenerate PCR. Additionally, sequences corresponding to *vasa *and *nanos *were subsequently identified from a large transcriptome sequencing project undertaken using 454 pyrosequencing technology (Smith, Benton, Hyink and Dearden, unpublished data). Sequences were assembled using CAP3 [42] and similarity to known *vasa *and *nos *sequences assessed using Blastx [43].

### Phylogenetic analysis

Phylogenetic analysis was carried out on predicted protein sequences for Vasa and Nanos aligned using ClustalX [44]. Bayesian phylogenetic analysis was carried out with the MrBayes software [45], using either the WAG (Vasa) [46] or BLOSUM (Nanos) models of amino acid substitution - after being identified as the appropriate model after initial experiments using mixed models. Twenty-five per cent of the initial trees were discarded as 'burnin' and the resulting consensus tree visualized using Dendroscope [47].

### *In situ *hybridization

Rotifers were harvested from culture medium by being sieved through a 74 μm filter (Sigma CD1 cell culture sieves). Rotifers were then resuspended in artificial seawater and relaxed with 10% ethanol for 5 min. Formaldehyde was then added to 4% and the rotifer fixed for 10-15 min. Rotifers were then washed twice in PTw (phosphate buffered saline + 0.1% Tween 20), transferred into a glass test tube and sonicated in a Benchtop Ultrasonic Cleaner (Model 80T, Soniclean, Thebarton, South Australia, http://www.soniclean.com.au; 60W pulse swept power output, fixed frequency of 33 +/-3 kHz) for 15-30 s. Rotifers were then allowed to settle and transferred to fresh PTw and incubated with 10 μg/μL proteinase K for 10-15 min at room temperature. The rotifers were washed in PTw twice, formaldehyde was added to 4% and then they were incubated at room temperature for 15 min. The tissue was then washed six times in PTw, transferred to hybridization buffer (50% formamide, 4 × standard saline citrate (SSC), 1 × Denhardt's solution, 250 *μ*g/mL yeast total RNA, 250 *μ*g/mL boiled salmon sperm or calf thymus DNA, 50 *μ*g/mL heparin (Sigma), 0.1% Tween 20, 5% dextran sulphate) and incubated for 2-3 h at 52°. Typically, hundreds of rotifer adults and embryos were used in each *in-situ *hybridization reaction in order to provide as broad a developmental time-course as possible.

Digoxigenin (DIG) labelled RNA probes for *in situ *hybridization were prepared as described in [48]. Two doses of 4 μL probe were digested in an equal volume of carbonate buffer at 60° for up to 30 min (depending on the length of the probe). One hundred microlitres of hybridization buffer was then added to the probe. The hybridization buffer was removed from the rotifers and replaced with the digested probe solution and the mixture incubated overnight at 56°. Rotifers were then washed seven times over 24 hours in 50% formamide, 2 × SSC, 0.1% Tween 20 at 56°.

Rotifers were transferred into PTw, rinsed three times and incubated in PTw + 0.1% w/v bovine serum albumin (BSA) for 15 min. The DIG hapten was detected using a 1:500 dilution of anti-DIG-alkaline phosphatase antibodies (Roche Applied Science, IN, USA) in PTw + 0.1% BSA for 90 min at room temperature. The antibody solution was removed and the rotifers washed six times in PTw over 2 h. Rotifers were washed twice in alkaline phosphatase (AP) staining buffer (100 mM Tris pH 9.5, 100 mM NaCl, 50 mM MgCl_2_, 0.1% Tween 20) and AP activity detected using 4.5 μL nitro blue tetrazolium chloride (75 mg/mL in dimethylformamide (DMF), Roche Applied Science) and 3.5 μL 5-bromo-4-chloro-3-indolyl-phosphate (50 mg/mL in DMF, Roche Applied Science) in AP buffer. Staining was monitored under a stereomicroscope and the tissues washed and transferred to methanol for de-staining when clear staining was observed. Tissues were rehydrated after the methanol wash, counterstained by incubation for 1 h in PTx containing either 1:1000 ProLong^® ^Gold antifade reagent with DAPI or 1 μM YOYO-1 (Invitrogen), cleared and mounted in 70% glycerol and examined under an Olympus BX61 compound microscope.

### Histochemistry

Rotifers were harvested, fixed and sonicated as described above for *in situ *hybridization. Rotifers were placed in DAB staining solution [49] + 0.8% NiCl_2_. After 5 min pre-incubation H_2_O_2 _was added to 0.006% and the staining was monitored under a dissecting microscope. Staining was stopped by washing in PTx and the specimens were counterstained by incubation for 1 h in PTx containing either 1:1000 ProLong^® ^Gold antifade reagent with DAPI, 1 uM propidium iodide or 1 μM YOYO-1 (Invitrogen). Rotifers were cleared and mounted in 70% glycerol and examined on an Olympus BX61 microscope.

## Abbreviations

AP: alkaline phosphatase; BSA: bovine serum albumin; DIC: differential interference contrast; DIG: digoxigenin; EST: expressed sequence tag; MAPK: mitogen-activated protein kinase; PCR: polymerase chain reaction; PGC: primordial germ cells; PTw: phosphaste buffered saline plus Tween.

## Competing interests

The authors declare that they have no competing interests

## Authors' contributions

JMS carried out some of the experiments, including molecular cloning, and contributed to drafting of the paper. AGC developed the techniques used in these analyses. PKD designed the study, carried out *in-situ *hybridization, immunohistochemistry and phylogenetics and contributed to the drafting the paper.

## Supplementary Material

Additional file 1 **Supplemental Figure 1**. Unrooted Phylogram of *Brachionus *16 S ribosomal sequences. Three 16 S sequences from the stock of rotifers used in this experiment cluster with *Brachionu*s *plicatilis *species against other *Brachionus *species and *Euchlanis dilatata*. Two *Brachionu*s species, one an isolate from Nevada with no species name in the database, and *B. manjavacas*, fall within the *plicatilis *clade. The rotifers used in this study cluster most closely with the Nevada isolate of *B. plicatilis*. Phylogram was constructed with MrBayes using the 4by4 model of nucleotide substitution. 25% of the initial trees were discarded as 'burnin' and the resulting consensus tree visualized using Dendroscope [47]. Click here for file
