# Supplementary figures and images for: Germ cell specification and ovary structure in the rotifer Brachionus plicatilis
Source: EvoDevo. 2010 Aug 2;1:5. doi: 10.1186/2041-9139-1-5 (PMC2938724; doi:10.1186/2041-9139-1-5)

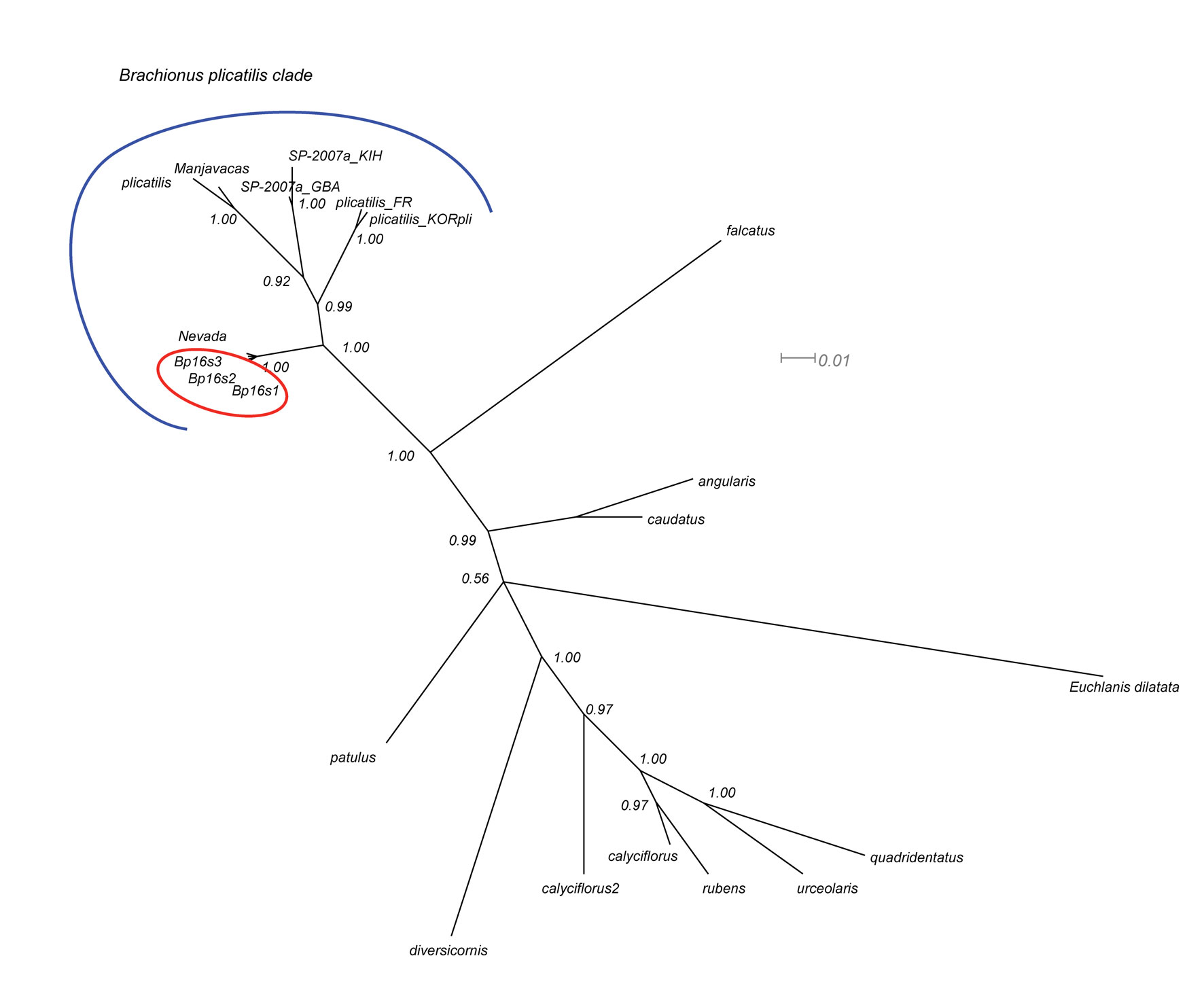

Supplement: Additional file 1 — Supplemental Figure 1. Unrooted Phylogram of Brachionus 16 S ribosomal sequences. Three 16 S sequences from the stock of rotifers used in this experiment cluster with Brachionus plicatilis species against other Brachionus species and Euchlanis dilatata. Two Brachionus species, one an isolate from Nevada with no species name in the database, and B. manjavacas, fall within the plicatilis clade. The rotifers used in this study cluster most closely with the Nevada isolate of B. plicatilis. Phylogram was constructed with MrBayes using the 4by4 model of nucleotide substitution. 25% of the initial trees were discarded as 'burnin' and the resulting consensus tree visualized using Dendroscope [47]. [file 2041-9139-1-5-S1.TIFF]
